# Applications of contact predictions to structural biology

**DOI:** 10.1107/S2052252517005115

**Published:** 2017-04-18

**Authors:** Felix Simkovic, Sergey Ovchinnikov, David Baker, Daniel J. Rigden

**Affiliations:** a Institute of Integrative Biology, University of Liverpool, Liverpool L69 7ZB, England; b Department of Biochemistry, University of Washington, Seattle, WA 98195, USA; c Institute for Protein Design, University of Washington, Seattle, WA 98195, USA; d Howard Hughes Medical Institute, University of Washington, Box 357370, Seattle, WA 98195, USA

**Keywords:** evolutionary covariance, predicted contacts, NMR distance restraints, X-ray crystallography, structural bioinformatics

## Abstract

Evolutionary pressure on residue interactions, intramolecular or intermolecular, that are important for protein structure or function can lead to covariance between the two positions. Recent methodological advances allow much more accurate contact predictions to be derived from this evolutionary covariance signal. The practical application of contact predictions has largely been confined to structural bioinformatics, yet, as this work seeks to demonstrate, the data can be of enormous value to the structural biologist working in X-ray crystallo­graphy, cryo-EM or NMR. Integrative structural bioinformatics packages such as *Rosetta* can already exploit contact predictions in a variety of ways. The contribution of contact predictions begins at construct design, where structural domains may need to be expressed separately and contact predictions can help to predict domain limits. Structure solution by molecular replacement (MR) benefits from contact predictions in diverse ways: in difficult cases, more accurate search models can be constructed using *ab initio* modelling when predictions are available, while intermolecular contact predictions can allow the construction of larger, oligomeric search models. Furthermore, MR using supersecondary motifs or large-scale screens against the PDB can exploit information, such as the parallel or antiparallel nature of any β-strand pairing in the target, that can be inferred from contact predictions. Contact information will be particularly valuable in the determination of lower resolution structures by helping to assign sequence register. In large complexes, contact information may allow the identity of a protein responsible for a certain region of density to be determined and then assist in the orientation of an available model within that density. In NMR, predicted contacts can provide long-range information to extend the upper size limit of the technique in a manner analogous but complementary to experimental methods. Finally, predicted contacts can distinguish between biologically relevant interfaces and mere lattice contacts in a final crystal structure, and have potential in the identification of functionally important regions and in foreseeing the consequences of mutations.

## Abstract 2

Recent developments allow the extraction of accurate contact predictions from multiple protein-sequence alignments. This review illustrates the manifold ways in which this information may assist the experimental structural biologist.

## 1. Introduction: contact predictions, their potential and their limitations   

The accurate prediction of residue contacts in proteins is a long-lasting challenge faced by the scientific community. Today, the prediction of residue contacts is usually performed using programs employing one or both of two algorithms: evolutionary coupling (EC) analysis and supervised machine learning (SML).

**Figure 1:**
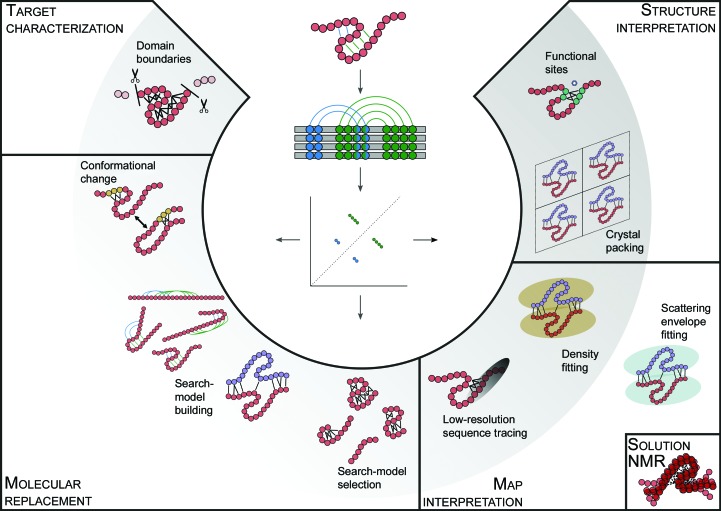
A schematic representation of the various points at which contact predictions, derived from multiple protein-sequence alignments (centre), are of use in the course (left to right) of structure determination by X-ray crystallography or cryo-EM. Applications to solution scattering data and NMR experiments are shown at the lower right.

EC methods use sequence information alone to identify the coordinated changes of residue pairs in protein families. These coordinated changes between participating residues are typically driven by the evolutionary pressure to preserve the structure and function of the protein. For many years, the prediction of contacting residue pairs by analysis of the coevolutionary pattern of amino acids in a protein family (Fig. 1 ▸) was limited by the statistical model applied. The so-called local statistical model was unable to distinguish accurate contact predictions, *i.e.* direct (*A*–*B* and *B*–*C*) covarying pairs of contacting residues, from indirect (*A*–*C*) pairs of residues that covary but are not in contact. A few years later, but largely forgotten until recently, Lapedes and coworkers were the first to apply a global statistical model to successfully overcome this hurdle (Lapedes *et al.*, 1999 ▸). More recently, various research groups revisited this concept and through different learning procedures of the same global model were able to improve the precision of the contact predictions dramatically. Whilst some rely on the principle of inverse covariance matrix estimation (Morcos *et al.*, 2011 ▸; Marks *et al.*, 2011 ▸; Jones *et al.*, 2012 ▸), it has been shown that pseudo-likelihood-based approaches result in the most accurate predictions (Balakrishnan *et al.*, 2011 ▸; Ekeberg *et al.*, 2013 ▸; Kamisetty *et al.*, 2013 ▸). However, these methods strongly rely on the availability of sufficient sequence homologues to be effective (see, for example, Morcos *et al.*, 2011 ▸; Kamisetty *et al.*, 2013 ▸; Jones *et al.*, 2015 ▸; Marks *et al.*, 2012 ▸; Ovchinnikov, Kim *et al.*, 2015 ▸; Skwark *et al.*, 2014 ▸). Nevertheless, together with the accelerating increases in the size of protein sequence databases (UniProt Consortium, 2015 ▸), these have made accurate contact prediction a reality (Marks *et al.*, 2012 ▸; de Juan *et al.*, 2013 ▸). Prominent contact-prediction methods and ancillary software, especially those available as servers, are listed in Table 1 ▸.

SML methods use a variety of sequence-dependent and sequence-independent information to predict contacting residue pairs (Cheng & Baldi, 2005 ▸; Shackelford & Karplus, 2007 ▸; González *et al.*, 2013 ▸; Wang & Xu, 2013 ▸; Zhang *et al.*, 2016 ▸; Du *et al.*, 2016 ▸). These methods derive contacts by analysing protein features, sequence profiles and mutual information. Although generally inferior to EC methods, SML algorithms can outperform EC algorithms for families with fewer homologous sequences (Skwark *et al.*, 2014 ▸; Wang & Xu, 2013 ▸; Ma *et al.*, 2015 ▸).

Since the SML methods learn and predict residue pairs at the same time, they suffer from a similar inability, as seen with older EC methods employing local statistical models, to distinguish directly and indirectly covarying residue pairs. However, to consider this potentially valuable information, more recent approaches combine methods from both categories: EC and SML. The SML predictions can be used as either priors to EC methods, one such example being *GREMLIN* (Ovchinnikov, Kinch *et al.*, 2015 ▸), or the output of multiple EC methods along with sequence profiles can be used as features in SML methods, for example *MetaPSICOV* (Jones *et al.*, 2015 ▸) and *PconsC*2 (Skwark *et al.*, 2014 ▸). Pipelines combining various EC and SML methods are often referred to as metapredictors, and a useful comparison of the best methods has recently been published (Wang *et al.*, 2017 ▸). In most cases, metapredictors outperform individual EC or SML methods in contact-prediction accuracy, but the improvement in structure prediction is less clear. The improvement in contact-prediction accuracy is particularly noticeable for cases where the available sequences are fewer or less diverse (de Oliveira *et al.*, 2016 ▸; Wuyun *et al.*, 2016 ▸). Major resources for contact prediction are listed in Table 1 ▸.

### 1.1. The number and diversity of sequences required for accurate contact predictions   

Alongside the earliest EC methods implementing a global statistical model, numerous guides have been proposed to approximate the minimum size of a multiple sequence alignment required for useful contact predictions. Originally, 1000 sequence homologues was considered to be the minimum for accurate predictions (Jones *et al.*, 2012 ▸; Marks *et al.*, 2012 ▸; Andreani & Söding, 2015 ▸). More recently, Marks and co­workers have recommended a more sequence-specific length-dependent factor for their method *EVFold*, whereby the sequence count in the alignment should exceed five times the protein length to obtain good *ab initio* folding results (Marks *et al.*, 2012 ▸). Others have also suggested similar requirements (Kamisetty *et al.*, 2013 ▸), but more recent work has slightly lowered this threshold by either improved covariance-detection algorithms (Ovchinnikov, Kinch *et al.*, 2015 ▸) or the use, where available, of structural information to decode a predicted contact map (Jeong & Kim, 2016 ▸). However, none of these estimates captures the diversity in a collection of sequences, which is also important for accurate contact prediction, and so they can be misleading. One of the most recent guidelines considers the diversity in a multiple sequence alignment after clustering at around 80% sequence identity: the number of effective sequences. Although different groups vary in their choice of sequence-identity cutoff (Morcos *et al.*, 2011 ▸; Jones *et al.*, 2015 ▸; Skwark *et al.*, 2014 ▸; Hopf *et al.*, 2012 ▸), the overall consensus suggests that the number of effective sequences is the most effective measure of alignment diversity and hence a useful predictor of prediction accuracy. The most recent EC methods require around 100–200 effective sequences for the top contact predictions to be accurate (Jones *et al.*, 2015 ▸; Skwark *et al.*, 2014 ▸). The number of contacts which can be accurately predicted increases with the number of effective sequences: to robustly generate accurate three-dimensional structure models requires roughly fivefold more sequences (Ovchinnikov *et al.*, 2017 ▸).

### 1.2. The challenge of distinguishing intramolecular and intermolecular contacts   

Currently, the methods used to predict residue-contact pairings cannot reliably distinguish intramolecular and intermolecular contacts, yet separating the two is important for the optimal performance of many of the methods mentioned below. Intramolecular residue contacts are generally more straightforward to predict, as a single protein-sequence alignment is sufficient. However, if the target forms homo-oligomers, the final contact prediction will potentially contain both intramolecular and intermolecular contacts if the latter are important for structure and function and hence under evolutionary pressure. Under such circumstances, interpretation of the predicted contacts can be misleading. At the same time, if structural information is available for the monomeric or dimeric protein structure, the predicted contact information can be essential in identifying potentially new oligomeric states by identifying strongly predicted but unsatisfied contact pairs (Hopf *et al.*, 2012 ▸; Jana *et al.*, 2014 ▸).

Where structural information to disentangle intramolecular and intermolecular contacts in homo-oligomers is not available, accessibility predictions may help: an intermolecular contact is more likely where both of a candidate contacting pair of residues are predicted to lie at the molecular surface. Already used in some contact-prediction algorithms to help the ranking of intramolecular contacts (for example *PconsC*2; Skwark *et al.*, 2014 ▸), the continued development of solvent-accessible surface-area prediction methods (Heffernan *et al.*, 2015 ▸) should facilitate the partitioning of intramolecular and intermolecular contacts in predicted contact maps. For transmembrane proteins, lipophilicity predictions are commonly used to detect membrane-facing residues (Koehler Leman *et al.*, 2015 ▸), which could help to distinguish intermolecular contacts in the bilayer.

In cases of hetero-oligomeric protein complexes, a different challenge is faced when predicting contact pairs. Although the disentanglement of intramolecular and intermolecular contacts is not required, the generation of the multiple sequence alignment for EC methods is challenging because orthologous interacting pairs of sequences must reliably be identified from a large number of species. Typically, sequences from individual alignments are paired using bacterial genome coordinates, *i.e.* the closer their location in the genome the more likely their co-expression and physical interaction (Ovchinnikov *et al.*, 2014 ▸; Hopf *et al.*, 2014 ▸; Skerker *et al.*, 2008 ▸). This information is important because the conservation of protein–protein interactions may not be present amongst all homologues. Using this approach, several studies have shown the applicability of EC methods to hetero-oligomeric protein complexes. For example, Hopf and coworkers correctly predicted 17 out of 19 residue contacts in the interface of the DinJ–YafQ complex (Hopf *et al.*, 2014 ▸). Ovchinnikov and coworkers reliably predicted the few contacting residue pairs between the proteins of the 50S ribosomal subunit complex and other protein complexes (Ovchinnikov *et al.*, 2014 ▸). In each case, the predicted contact information enabled accurate models of the protein complexes to be determined based on the individual component structures. A second, simpler method has been developed to create sequence alignments for protein–protein interface contact predictions. This method is bacterial genome-independent and matches orthologous sequence pairs using genome *BLAST* scores (Iserte *et al.*, 2015 ▸; Yu *et al.*, 2016 ▸; Ochoa & Pazos, 2010 ▸). In general, this second alignment-generation method may produce a less accurate set of matched pairs for prokaryotic proteins, but has already proven to be useful for protein–protein interactions in eukaryotes (Iserte *et al.*, 2015 ▸), and new methods may well improve the accuracy for these organisms (Gueudré *et al.*, 2016 ▸; Bitbol *et al.*, 2016 ▸). The reader is referred to Table 1 ▸ for major resources in this area.

## 2. Predicting the domain structure of the target   

Protein domains are the units of folded protein structure. An accurate accounting of the domain composition of a protein as a potential subject of structural characterization is valuable before target selection, when sample preparation is being planned, and when considering strategies for protein crystal structure solution. Accurately defined domain boundaries improve the performance of various key bioinformatics methods, such as tools that recognize distant homology between (part of) the target and known protein structures (Rigden, 2002 ▸), *ab initio* modelling (see, for example, Baker *et al.*, 2016 ▸) and even contact prediction itself (Kosciolek & Jones, 2015 ▸). The recognition of nontrivial evolutionary relationships by sensitive sequence comparisons or (contact-aided) *ab initio* modelling may help the structural biologist at the early stage of assessing the inherent novelty or otherwise of a new putative target. Commonly, proteins are expressed heterologously in an incomplete form, especially for NMR or X-ray crystallographic studies. There are various reasons for this. For example, intrinsically disordered terminal regions, which are known to impede crystallization (Slabinski *et al.*, 2007 ▸), would typically be eliminated from a protein destined for crystallization. Very large proteins, the expression and purification of which are likely to prove difficult, will generally be dealt with in sections commencing and terminating at domain boundaries (see, for example, Zacharchenko *et al.*, 2015 ▸). Finally, as a methodology that is limited in its tractable molecular-weight range, NMR studies often focus on isolated domains.

Predicting domain boundaries using predicted contacts (Fig. 1 ▸) is based on a very simple idea: that native contacts, and hence predicted contacts, are more abundant within domains than between domains. Thus, in a contact map for a protein of two equally sized domains, the area containing predicted contacts between residues in the first half and residues in the second half would be sparsely populated in comparison to the zones containing intra-domain predictions. These patterns are often apparent on visual inspection, and have been employed by bioinformaticians to parse target sequences for *ab initio* modelling (Ovchinnikov, Kim *et al.*, 2015 ▸; Baker *et al.*, 2016 ▸), but can also be analysed quantitatively. For this, putative domain boundaries are sampled along the protein chain, with stronger predictions corresponding to minima in the density of predicted interdomain contacts (Rigden, 2002 ▸). Dating from an epoch of lower quality predictions, the idea has been revisited recently and implemented using a kernel-smoothing method (Sadowski, 2013 ▸). A comparison with other methods of sequence-based domain-boundary prediction showed it to be the best performing and also to be applicable to proteins containing more than two domains (Sadowski, 2013 ▸). That *domainpred* software would be the recommended approach for structural biologists interested in predicting the domain composition of their proteins of interest, but it seems currently unavailable. However, similar functionality has been made available in *ConKit* (Table 1 ▸) which can accept the required list of predicted contacts in a wide variety of formats. Finally, interesting recent work has also demonstrated the ability of covariance analysis to detect putative folding units within largely intrinsically disordered proteins (Toth-Petroczy *et al.*, 2016 ▸).

## 3. Applications to crystal structure determination   

For protein crystal structure solution the phasing problem – the ability to only directly measure intensity data – must be overcome using experimental or computational means. Contact prediction is most relevant to molecular replacement (MR) as a computational route to structure solution (Fig. 1 ▸). In MR, a ‘search model’ that is believed to approximate at least a part of the unknown target structure is positioned in the symmetric unit, usually by sequential rotation and translation steps. This placed structure can then be used as a source of approximate phase information, allowing the calculation of initial electron-density maps. However, before considering MR specifically it is worth reiterating the value, for all phasing approaches, of a comprehensive understanding of the domain structure of the protein target.

### 3.1. Better characterizing the target   

Recognized evolutionary relationships between (domains of) the target and known protein structures or families can valuably predict the existence of features facilitating experimental structure solution. For example, metal-binding, base-binding or cofactor-binding sites can each ligate natural ligands, or artificial analogues thereof, containing atoms with useful anomalous scattering properties and/or high masses. Single-crystal or multi-crystal diffraction data in such cases are suitable for solution by anomalous scattering and/or isomorphous replacement approaches (Dauter, 2002 ▸; Hendrickson, 2014 ▸). Prominent methods for detecting even distant homologies include *HHpred* (Söding *et al.*, 2005 ▸; https://toolkit.tuebingen.mpg.de/hhpred), *Phyre* (Kelley *et al.*, 2015 ▸; http://www.sbg.bio.ic.ac.uk/~phyre2/) and *FFA*S-3*D* (Xu *et al.*, 2014 ▸; http://ffas.godziklab.org). Such methods to recognize hidden evolutionary relationships between the target and known structures benefit from contact prediction in two ways. Firstly, the improved domain parsing described above can improve the sensitivity of homology-detection tools: known folds or families can be more confidently matched to sub­sections of the target encompassing individual domains than they can to a whole multi-domain sequence (Rigden, 2002 ▸). Secondly, fold-recognition methods may, in the near future, be able to directly exploit predicted contact information: putative matched folds that are in accord with the predicted contacts for the target, according to the alignment of the two, can be awarded a higher score. Work in this area has recently been published (Ovchinnikov *et al.*, 2017 ▸; Taylor, 2016 ▸).

### 3.2. Deriving and ranking search models for MR   

Predicted contacts can help to derive better search models by informing on the super-secondary, tertiary and quaternary structure of the target (Fig. 1 ▸). Perhaps the most obvious application lies in using contact predictions to build better structure models. Structural bioinformaticians have been quick to exploit predicted contact information to model representatives of structurally uncharacterized protein families (Ovchinnikov, Kinch *et al.*, 2015 ▸; Hopf *et al.*, 2012 ▸). While the results are typically sufficient for very valuable functional inference by fold matching (Ovchinnikov, Kinch *et al.*, 2015 ▸), the overall moderate accuracy of the final models, compounded in some cases by poor backbone stereochemistry (Marks *et al.*, 2011 ▸), left open the question as to their value to MR. Addressing this issue, Simkovic and coworkers recently explored the value of contact-assisted *ab initio* models in the context of the *AMPLE* cluster-and-truncate search-model preparation framework (Simkovic *et al.*, 2016 ▸). The work compared unassisted models, those informed by the predictions from the general method *PconsC*2 (Skwark *et al.*, 2014 ▸), and those guided by a novel combination of *PconsC*2 with a β-sheet-specific method, *bbcontacts* (Andreani & Söding, 2015 ▸). In a set of 21 cases, spanning sizes of 62–221 residues, resolutions of 1.0–2.3 Å and all fold classes, they found multiple targets that could only be solved using models informed by predicted contact information. The benefits of this information were twofold: better modelling of larger proteins extended the upper size limit of the method, and β-rich proteins, which were previously very rarely successful (Bibby *et al.*, 2012 ▸), were successfully solved more frequently. A quite independent relevance of predicted contacts to *AMPLE*’s search-model preparation comes from the realisation that predicted contacts, rather like sequence conservation, derive from evolutionary pressure to retain biologically important structural features (see §[Sec sec6]6). Thus, predicted contacts may help to identify the key features shared between a target that are known or suspected to be only distantly related to deposited structures. The ability of contact predictions to guide search-model preparation, even of single homologues, using *AMPLE*’s truncation approach is currently being explored.

Finally, for tertiary structure, intriguing recent work points to a general ability of contact predictions to enable predictions to be made about alternative conformations of a given structure (Jana *et al.*, 2014 ▸; Sfriso *et al.*, 2016 ▸). The rationale here is that any biologically important conformation will lead to evolutionary pressure on relevant contacts that would manifest itself as a detectable covariance between the pair of positions involved. This phenomenon was noted previously during contact-based modelling, where a single modelled structure proved incapable of explaining fully the pattern of covarying residue pairs since the predicted contacts resulted from two distinct conformations (Hopf *et al.*, 2012 ▸). This opens the way to convert a single structure of a homologue of the template to a set of putative conformations (Sfriso *et al.*, 2016 ▸) to trial by MR. This might enable successful structure solution in frequently encountered cases where a protein exhibits structural plasticity (open and closed forms, R- and T-state *etc.*) yet the target crystal is not in a conformation represented by the PDB.

The availability of the *bbcontacts* algorithm (Andreani & Söding, 2015 ▸), which can sensitively detect and distinguish parallel and antiparallel β-sheet predictions in a predicted contact map, also offers a route to search-model ranking for library-based MR methods (Fig. 1 ▸). Such programs include *ARCIMBOLDO*–*BORGES*, which attempts structure solution using libraries of recurring super-secondary structures composed of a few regular secondary-structure elements derived from an analysis of the PDB (Sammito *et al.*, 2013 ▸). These libraries are relatively large, but the runtimes for structure solution could be reduced by assigning parallel and/or antiparallel β-sheets to the target and ordering the processing of search models to prioritize those containing the correct kind of strand matching. Similarly, approaches based on screening of the whole PDB (Keegan *et al.*, 2016 ▸; Stokes-Rees & Sliz, 2010 ▸) may also rank search models according to the predicted β-sheet composition of the target.

Predicting the quaternary structure of the target may also be valuable for MR and is relevant for both homo-oligomers and hetero-oligomers (Fig. 1 ▸). In essence, the data-driven docking approaches developed in structural bioinformatics to exploit predicted contact information can be used to derive and rank oligomeric search models. In comparison to individual subunits, these contain a greater fraction of the scattering matter of the target and therefore, if sufficiently accurate, should exhibit improved signal to noise and hence a better chance of successful structure solution. A single docking server, *InterEvDock*, that automatically incorporates evolutionary covariance into its calculations has very recently become available (Yu *et al.*, 2016 ▸; see Table 1 ▸). It carries out rigid-body docking of two structures using *FRODOCK* (Ramírez-Aportela *et al.*, 2016 ▸). A pool of 10 000 poses is then scored in three different fashions, one being a residue-based co-evolution score derived from the *i-COMS* server (Iserte *et al.*, 2015 ▸; Ochoa & Pazos, 2010 ▸; Table 1 ▸), and the server reports the top ten consensus models found by clustering the best scoring poses by each of the three evaluations. A crystallo­grapher might also reproduce approaches in which predicted contacts either guide docking (Hopf *et al.*, 2014 ▸) with *HADDOCK* (Dominguez *et al.*, 2003 ▸) or rank the results of docking with *PatchDock* v.1.0 (Duhovny *et al.*, 2002 ▸) and refine with *Rosetta* (Ovchinnikov *et al.*, 2014 ▸). At present, most docking servers are not optimized to exploit predicted contact information: they may accept sets of residues on each docked protein believed to be close to the interface, but do not accept paired predicted contacts. Although this can be expected to change in the near future, a user would currently be obliged to inspect the results manually to determine whether high-ranking intermolecular contacts are present in poses from some top-performing servers such as *ClusPro* (Comeau *et al.*, 2004 ▸) or servers specialized for the flexible docking of protein partners such as *SwarmDock* (Torchala *et al.*, 2013 ▸). Finally, it is worth reiterating here the additional difficulties of contact prediction between two different proteins: a concatenated alignment in which orthologues of each are matched between a series of species is required. Reliable identification of such pairs is not trivial. For this reason, some current leading methods such as *GREMLIN* (Ovchinnikov *et al.*, 2014 ▸) have thus far focused on cases in which microbial genome-context information provides additional support for orthologue identification. Of course, for contacts in homo-oligomers these limitations do not apply. Prediction of these assemblies will be particularly reliable in cases such as membrane pores, where symmetry provides an additional useful restraint on docking (see, for example, DiMaio, Leaver-Fay *et al.*, 2011 ▸).

## 4. Fitting structures and tracing sequences in lower resolution maps and envelopes   

The outcomes of structural biology methods that aim to yield atomic models, such as X-ray crystallography and, increasingly, cryo-EM, depend sensitively on the data available. Only at ultrahigh resolution can X-ray structures be accurately refined using the X-ray diffraction data alone since the data-to-parameter is too low (Rupp, 2009 ▸). More typically, the refinement of crystal structures employs additional information to supplement the observed diffraction data, most obviously chemical information such as bond distances but also, where available, additional restraints from noncrystallographic symmetry. At lower resolutions, however, even this additional information may prove to be inadequate for atomic refinement, and reconstructions may therefore comprise only structures or models for individual subunits or domains placed within a low-resolution map or envelope and rigid-body refined. It is in the area of lower resolution structure interpretation that contact predictions have the most to offer by providing additional restraints that should be satisfied by the emerging structural model (Fig. 1 ▸). These predictions will therefore help not only medium- to low-resolution crystal structures and cryo-EM reconstructions, but also the interpretation of envelopes derived from SAXS and SANS (Svergun *et al.*, 2013 ▸). Applications can be divided into those dependent on intramolecular contact predictions and those deriving from intermolecular restraints.

Intramolecular contacts are valuable here in several ways, as already mentioned. For novel folds for which low-resolution data are available, contacts will enable better models to be derived for subsequent fitting into maps or envelopes. These would be cases such as ribosomal structures (see, for example, Brown *et al.*, 2014 ▸), where supernumerary subunits could be modelled *ab initio* and fitted using the approach mentioned above. In a more recent study, *Rosetta*-generated *ab initio* models, guided by evolutionary restraints, were used to resolve the amino-acid registry, the connectivity of the helices and the placement of the subunits of the cytochrome *bd* oxidase complex in a low-resolution (3.1–4 Å) electron-density map derived from weak experimental phase information (Safarian *et al.*, 2016 ▸). In cases where a structure, experimental or modelled, cannot be fitted well to the map or envelope, the prediction of alternative conformations using predicted contacts (Sfriso *et al.*, 2016 ▸) may produce better fitting candidate structures.

The assignment of sequence register to a low-resolution, backbone-traced structure is another potential area of application (Fig. 1 ▸). Programs such as *Buccaneer* (Cowtan, 2006 ▸) and *ARP*/*wARP* (Langer *et al.*, 2008 ▸) recognize side-chain density shape and attempt to dock putatively assigned residues to a provided sequence. However, below a certain resolution the number of assigned residues and the confidence of their identification will drop. At this point contact predictions may help: a strong prediction from one residue that is already docked to the sequence to another ill-defined position may anchor sequence-register definition for a whole range of the target protein. One example of such an application is the successful tracing of the protein sequence of the a subunit of *Thermus thermophilus* V/A-ATPase in a 6.4 Å resolution cryo-EM density map, which resulted in a complete model of the rotary ATPase (Schep *et al.*, 2016 ▸). Covariance analysis was also used to confirm the helical assignments of the 2.95 Å resolution crystal structure of a human tetraspanin (Zimmerman *et al.*, 2016 ▸). There is therefore a need to make predicted contact information conveniently available from within structure-building and refinement programs.

As mentioned above, predicted intermolecular contacts offer a generic way to rank and select the most likely interaction mode of a pair of structures (Ovchinnikov *et al.*, 2014 ▸; Hopf *et al.*, 2014 ▸; Yu *et al.*, 2016 ▸). The most obvious application is therefore to assist in the interpretation of density for multi-subunit complexes. Proteins that are significantly anisotropic can often be fitted quite reliably even at lower density, but three-dimensional forms with fewer features often fit equally well to a map or envelope in several ways (Joseph *et al.*, 2016 ▸). Disambiguating these situations using sequence conservation has recently been explored (Joseph *et al.*, 2016 ▸), but predicted contacts arguably offer a more direct signal of intermolecular interaction and are independent of existing interaction information (Segura *et al.*, 2016 ▸). For example, in the cytochrome *bd* oxidase work mentioned above, covariation information was used to confirm the intermolecular interactions resulting from the placement of the covariance-assisted *ab initio* models (Safarian *et al.*, 2016 ▸). In the future, such information could be used in several fashions. Use might first entail the prior generation of range of potential multimeric structures, each in broad agreement with the predicted contact signal, in the expectation that one might fit much better than the others. Such a library could also be used for direct fitting to experimental scattering information (see, for example, Schindler *et al.*, 2016 ▸; Jimenez-Garcia *et al.*, 2015 ▸). Secondly, putative fits for a first subunit could be visually inspected for those that are compatible with the placement of the second subunit in such a way as to satisfy the predicted contacts. Thirdly, programs for the automated fitting of structures to density such as *gamma-TEMPy* (Pandurangan *et al.*, 2015 ▸) or 3*DIANA* (Segura *et al.*, 2016 ▸) could be engineered to directly include satisfaction of predicted contact information in their scoring functions. More speculatively, predicted contacts may ultimately inform not just on the orientation of the known subunits in a complex but also on the composition of a complex, information that may be only incompletely available. Thus, future genome-scale screening to find which proteins share covarying residue pairs with which others, and thereby assemble an *in silico* interactome, has already been envisaged (Hopf *et al.*, 2014 ▸). Such information might help the structural biologist synthesize, purify and reconstruct all necessary components of the stable, biologically relevant macromolecular complex.

## 5. Nuclear magnetic resonance   

NMR is a method in which the use of labelling strategies to provide additional restraints, particularly long-range distance restraints, to guide folding has been key to extending the upper bound on tractable molecular weight to larger proteins (Raman *et al.*, 2010 ▸; Lange *et al.*, 2012 ▸; Göbl *et al.*, 2014 ▸). For RNA and protein–RNA complexes, additional restraints have been derived from EPR information (Duss *et al.*, 2014 ▸, 2015 ▸), and fluorescence can also provide distance restraints (Göbl *et al.*, 2014 ▸). However, researchers have also been quick to perceive the value of predicted contacts derived from evolutionary covariance (Tang *et al.*, 2015 ▸) which, in comparison to experimental methods, avoid complications relating to the labelling of the macromolecule (Duss *et al.*, 2015 ▸) and to any modification-induced change to the structure, dynamics or function of the target. The major effort in the area so far is *EC-NMR* (Tang *et al.*, 2015 ▸), in which *CYANA* is used to generate structural ensembles based on both NMR data (NMR resonance assignments for ^1^H–^15^N and/or ^1^H–^13^C methyl resonances and NOESY cross-peaks) and covariance-based predicted contacts. These ensembles are then used in an iterative fashion to revisit and edit the input data, with the one data type providing an internal check on the other, enabling the elimination of incorrect NOESY peaks and false-positive contact predictions. The method thus elegantly exploits the complementarity of the two data sources, ultimately producing structures based on refined and improved input data sets. Contact-prediction information can also be fed into *CS-Rosetta* since *Rosetta*’s sampling and scoring functions have proven to be highly effective for structure determination by NMR (Raman *et al.*, 2010 ▸; van der Schot & Bonvin, 2015 ▸).

## 6. Structural analysis and interpretation   

When analysing a refined crystal structure, it is not always straightforward to distinguish biologically meaningful interactions between subunits from those intermolecular contacts that simply result from the formation of a crystal lattice (Capitani *et al.*, 2016 ▸). Years of research suggest that no single metric of interfaces can partition physiologically relevant interactions from mere crystal contacts (Jones & Thornton, 1996 ▸), so that current state-of-the-art approaches such as *jsPISA* offer multiple relevant measurements such as interface area, hydrophobicity and predicted binding energy (Krissinel, 2015 ▸). Contact predictions offer a further appealing way to distinguish the two interface classes (Fig. 1 ▸) since, as mentioned at the outset, evolutionary covariance spanning an interface will only emerge where pressure to maintain the interaction has been exerted during evolution. Although complications will emerge when, for example, homologous proteins genuinely differ in their oligomeric state, notably successful use of contact predictions to help parse crystal structure contents has already been seen with structures of protocadherin domain fragments (Nicoludis *et al.*, 2015 ▸). There, contact predictions supported certain interfaces as biologically relevant over others of similar size and performance with respect to conventional metrics, in a fashion also supported by sequence conservation and the positions of post-translational modification sites.

Structural bioinformatics provides a wide variety of orthogonal analyses that can help to predict the location of functional sites in a given structure (Rigden, 2017 ▸). The density of the covariance signal across the structure, coded for example as an EC score reflecting the number and the strength of the contact predictions associated with each residue, has the potential to be a useful addition to the list (Fig. 1 ▸). For example, in work using contact predictions to fold transmembrane proteins, residues with high scores were found at known substrate-binding sites (Hopf *et al.*, 2012 ▸). Similarly, in models of families that have not yet been structurally characterized experimentally, high-scoring residues were found at predicted catalytic or cofactor-binding sites and lining probable pores (Hopf *et al.*, 2012 ▸). More recent work takes a network approach to infer functional sites from contact predictions (Parente *et al.*, 2015 ▸). One example is the identification of functional residues, both catalytic and interfacial, in the aldolase family using eigenvector centrality, which describes residue hotspots in contact maps (Parente *et al.*, 2015 ▸). A very recent paper exploits a known structure to aid the interpretation of contact-prediction information, enabling functional site prediction (Jeong & Kim, 2016 ▸). Another study uses contact predictions to identify druggable protein–protein interfaces through a combination of fragment docking and EC methods (Bai *et al.*, 2016 ▸), and a further recent paper demonstrates the value of the covariance signal for inferring the detrimental or benign nature of single amino-acid polymorphisms (Hopf *et al.*, 2016 ▸). Finally, it is interesting to view longstanding conventional sequence conservation (Ashkenazy *et al.*, 2016 ▸) and the new pairwise covariance methods discussed here as the simplest cases of coevolutionary analysis, an analysis which can readily be extended to determine larger functionally relevant covarying groups (Grigolon *et al.*, 2016 ▸).

## 7. *Rosetta* as a unifying structural bioinformatics framework   

The utility of contact predictions can be compared with that of experimental methods for deriving distance restraints, such as chemical cross-linking (Belsom *et al.*, 2016 ▸), spin-labelling combined with electron paramagnetic resonance (Fischer *et al.*, 2016 ▸) or fluorescence (Göbl *et al.*, 2014 ▸), which have played such a valuable role in the integrative structure determination of large complexes (Webb *et al.*, 2011 ▸). Recent blind analysis of the value of experimental cross-linking data to protein structure prediction (Belsom *et al.*, 2016 ▸) has highlighted the limitations of uneven coverage and poor definition of β-sheets. Selective labelling to gain long-range distance information for NMR of large proteins also suffers from its own complications (Lange *et al.*, 2012 ▸) when the methyl-containing probe residues are unevenly distributed. Although contact prediction has its own limitations, it is well placed to occupy a complementary role to experimental distance restraints (Tang *et al.*, 2015 ▸). Effectively exploiting contact predictions alongside sources of experimental restraints for structure prediction requires an extensible and unifying structural bioinformatics approach. Here, it is worth considering *Rosetta* in more detail as a software package that is well suited to this rationale and has a considerable track record in this area.

Macromolecular structure-prediction approaches such as *Rosetta* are based on the hypothesis that the native states of proteins are at global free-energy minima, and carry out a large-scale search of conformational space for the lowest energy structure. The success of such approaches depends on two factors: the accuracy of the energy function and the ability of the search to converge on the lowest energy state. Because of the very large number of degrees of freedom in biomolecular systems, the second challenge, the search problem, is the primary bottleneck to accurate prediction. For all but the smallest proteins (less than 80 amino acids), the conformational space is too large for accurate *ab initio* structure prediction. However, when experimental information is available it can be used to focus the search for lowest energy states on the relevant part of the conformational search and can enable the determination of the structures of quite complex proteins and biomolecular complexes. For example, the incorporation of even quite limited electron-density data (DiMaio, Terwilliger *et al.*, 2011 ▸; DiMaio *et al.*, 2013 ▸), NMR data (Raman *et al.*, 2010 ▸; van der Schot & Bonvin, 2015 ▸) or cryo-EM data (DiMaio *et al.*, 2015 ▸; Wang *et al.*, 2015 ▸) into *Rosetta* can allow the generation of very accurate models. In contrast to conventional structure-prediction methods, the experimental data do not fully determine the structure – instead they guide the search process – and hence fewer data are required. Co-evolution data are treated within *Rosetta* just as experimental data are, and the power of co-evolution restraints to guide the search for the lowest energy structures has been illustrated in multiple quite accurate blind predictions (Ovchinnikov, Kim *et al.*, 2015 ▸; Safarian *et al.*, 2016 ▸). Within this framework, the integration of co-evolution data with cryo-EM, X-ray or NMR data is straightforward: all are read into *Rosetta* and used to guide the conformational search. The issue of how to weight the different sources of information (co-evolution data *versus* experimental data) guiding the search can be resolved by experimenting with different weightings and choosing that which results in models with the lowest energy.

## 8. Conclusion   

As we have shown, predicted contacts deriving from evolutionary covariance already offer exciting possibilities to the experimental structural biologist as much as to the bioinformatician. The area remains highly active and new approaches (see, for example, Yang *et al.*, 2016 ▸) can confidently be expected to continue to improve performance in the near future. These include approaches where additional information can be exploited to improve the precision of contact predictions (Zhang *et al.*, 2016 ▸; Hopf *et al.*, 2012 ▸; Wang & Barth, 2015 ▸; Hönigschmid & Frishman, 2016 ▸). Other recent progress has been made in the prediction of interacting pairs of proteins, from among paralogous families, without the help of genome-context information, developments which should increase the reach of intermolecular contact prediction still further (Gueudré *et al.*, 2016 ▸; Bitbol *et al.*, 2016 ▸).

## Figures and Tables

**Table 1:** Key methods in contact prediction or its application available as servers or downloads

Name of method	Description	Availability	URL	Citation
*HHblits*	Sequence-alignment generation by database search	Web server and local installation	https://toolkit.tuebingen.mpg.de/hhblits	Remmert *et al.* (2011 ▸)
*Jackhmmer*	Sequence alignment generation by database search	Web server and local installation	https://www.ebi.ac.uk/Tools/hmmer/search/jackhmmer	Johnson *et al.* (2010 ▸)
*CCMpred*	Contact-prediction application	Local installation	https://github.com/soedinglab/CCMpred	Seemayer *et al.* (2014 ▸)
*MetaPSICOV*	Intramolecular contact-prediction server	Web server and local installation	http://bioinf.cs.ucl.ac.uk/MetaPSICOV	Jones *et al.* (2015 ▸)
*GREMLIN*	Intramolecular and intermolecular contact-prediction server	Web server and local installation	http://gremlin.bakerlab.org	Ovchinnikov, Kinch *et al.* (2015 ▸)
*RaptorX-Contact*	Applies an ultradeep learning model to predict contacts: one of the best methods in CASP12	Web server and local installation	http://raptorx.uchicago.edu/ContactMap/	Wang *et al.* (2017 ▸)
*EVfold*	Intramolecular and intermolecular contact-prediction server with optional *ab initio* structure prediction	Web server	http://evfold.org/evfold-web/evfold.do	Marks *et al.* (2011 ▸)
*CONFOLD*	*Ab initio* structure-prediction server that takes input contacts	Web server	http://protein.rnet.missouri.edu/confold/	Adhikari *et al.* (2015 ▸)
*ConKit*	Python interface to contact prediction, visualization and evaluation with command-line scripts available	Local installation	http://www.conkit.org	Simkovic *et al.* (2017 ▸)
*ConEVA*	Contact-prediction evaluation server	Web server	http://cactus.rnet.missouri.edu/coneva/	Adhikari *et al.* (2016 ▸)
*MSAVOLVE*	*MATLAB* toolbox that includes numerous contact-prediction and related algorithms	Local installation	http://146.9.23.191/~gatti/coevolution/msavolve---simulation-and.html	Gatti (2015 ▸)
*Domainpred*	Perl scripts using kernel density estimation to parse domains from a list of predicted contacts	Local installation	Not currently available, but similar functionality is available in *ConKit*	Sadowski (2013 ▸)
*i-COMS*	*Interprotein COrrelated Mutations Server*: a webserver to calculate correlated mutations between proteins	Web server	http://i-coms.leloir.org.ar/index.php	Iserte *et al.* (2015 ▸)
*InterEvDock*	Protein–protein binding mode prediction server that uses contact predictions to help score poses	Web server	http://mobyle.rpbs.univ-paris-diderot.fr/cgi-bin/portal.py#forms::InterEvDock	Yu *et al.* (2016 ▸)

## References

[bb1] Adhikari, B., Bhattacharya, D., Cao, R. & Cheng, J. (2015). *Proteins*, **83**, 1436–1449. 10.1002/prot.24829 PMC4509844 25974172

[bb2] Adhikari, B., Nowotny, J., Bhattacharya, D., Hou, J. & Cheng, J. (2016). *BMC Bioinformatics*, **17**, 517. 10.1186/s12859-016-1404-z PMC5142288 27923350

[bb3] Andreani, J. & Söding, J. (2015). *Bioinformatics*, **31**, 1729–1737. 10.1093/bioinformatics/btv041 25618863

[bb4] Ashkenazy, H., Abadi, S., Martz, E., Chay, O., Mayrose, I., Pupko, T. & Ben-Tal, N. (2016). *Nucleic Acids Res.* **44**, W344–W350. 10.1093/nar/gkw408 PMC4987940 27166375

[bb5] Bai, F., Morcos, F., Cheng, R. R., Jiang, H. & Onuchic, J. N. (2016). *Proc. Natl. Acad. Sci. USA*, **113**, E8051–E8058. 10.1073/pnas.1615932113 PMC5167203 27911825

[bb6] Baker, J. A., Simkovic, F., Taylor, H. M. & Rigden, D. J. (2016). *Proteins*, **84**, 1431–1442. 10.1002/prot.25088 PMC5031224 27318187

[bb7] Balakrishnan, S., Kamisetty, H., Carbonell, J. G., Lee, S. & Langmead, C. J. (2011). *Proteins*, **79**, 1061–1078. 10.1002/prot.22934 21268112

[bb8] Belsom, A., Schneider, M., Brock, O. & Rappsilber, J. (2016). *Trends Biochem. Sci.* **41**, 564–567. 10.1016/j.tibs.2016.05.005 PMC4929974 27242194

[bb9] Bibby, J., Keegan, R. M., Mayans, O., Winn, M. D. & Rigden, D. J. (2012). *Acta Cryst.* D**68**, 1622–1631. 10.1107/S0907444912039194 23151627

[bb10] Bitbol, A. F., Dwyer, R. S., Colwell, L. J. & Wingreen, N. S. (2016). *Proc. Natl Acad. Sci. USA*, **113**, 12180–12185. 10.1073/pnas.1606762113 PMC5087060 27663738

[bb11] Brown, A., Amunts, A., Bai, X.-C., Sugimoto, Y., Edwards, P. C., Murshudov, G., Scheres, S. H. W. & Ramakrishnan, V. (2014). *Science*, **346**, 718–722. 10.1126/science.1258026 PMC4246062 25278503

[bb12] Capitani, G., Duarte, J. M., Baskaran, K., Bliven, S. & Somody, J. C. (2016). *Bioinformatics*, **32**, 481–489. 10.1093/bioinformatics/btv622 PMC4743631 26508758

[bb13] Cheng, J. & Baldi, P. (2005). *Bioinformatics*, **21**, i75–i84. 10.1093/bioinformatics/bti1004 15961501

[bb14] Comeau, S. R., Gatchell, D. W., Vajda, S. & Camacho, C. J. (2004). *Bioinformatics*, **20**, 45–50. 10.1093/bioinformatics/btg371 14693807

[bb15] Cowtan, K. (2006). *Acta Cryst.* D**62**, 1002–1011. 10.1107/S0907444906022116 16929101

[bb16] Dauter, Z. (2002). *Curr. Opin. Struct. Biol.* **12**, 674–678. 10.1016/s0959-440x(02)00372-x 12464322

[bb17] DiMaio, F., Echols, N., Headd, J. J., Terwilliger, T. C., Adams, P. D. & Baker, D. (2013). *Nat. Methods*, **10**, 1102–1104. 10.1038/nmeth.2648 PMC4116791 24076763

[bb18] DiMaio, F., Leaver-Fay, A., Bradley, P., Baker, D. & André, I. (2011). *PLoS One*, **6**, e20450. 10.1371/journal.pone.0020450 PMC3120754 21731614

[bb19] DiMaio, F., Song, Y., Li, X., Brunner, M. J., Xu, C., Conticello, V., Egelman, E., Marlovits, T. C., Cheng, Y. & Baker, D. (2015). *Nat. Methods*, **12**, 361–365. 10.1038/nmeth.3286 PMC4382417 25707030

[bb20] DiMaio, F., Terwilliger, T. C., Read, R. J., Wlodawer, A., Oberdorfer, G., Wagner, U., Valkov, E., Alon, A., Fass, D., Axelrod, H. L., Das, D., Vorobiev, S. M., Iwaï, H., Pokkuluri, P. R. & Baker, D. (2011). *Nature (London)*, **473**, 540–543. 10.1038/nature09964 PMC3365536 21532589

[bb21] Dominguez, C., Boelens, R. & Bonvin, A. M. J. J. (2003). *J. Am. Chem. Soc.* **125**, 1731–1737. 10.1021/ja026939x 12580598

[bb22] Du, T., Liao, L., Wu, C. & Sun, B. (2016). *Methods*, **110**, 97–105. 10.1016/j.ymeth.2016.06.001 27282356

[bb23] Duhovny, D., Nussinov, R. & Wolfson, H. J. (2002). *Algorithms in Bioinformatics*, edited by R. Guigó & D. Gusfield, pp. 185–200. Berlin, Heidelberg: Springer-Verlag. https://doi.org/10.1007/3-540-45784-4_14.

[bb24] Duss, O., Yulikov, M., Allain, F. H.-T. & Jeschke, G. (2015). *Methods Enzymol.* **558**, 279–331. 10.1016/bs.mie.2015.02.005 26068745

[bb25] Duss, O., Yulikov, M., Jeschke, G. & Allain, F. H.-T. (2014). *Nat. Commun.* **5**, 3669. 10.1038/ncomms4669 24828280

[bb26] Ekeberg, M., Lövkvist, C., Lan, Y., Weigt, M. & Aurell, E. (2013). *Phys. Rev. E*, **87**, 012707. 10.1103/PhysRevE.87.012707 23410359

[bb27] Fischer, A. W., Bordignon, E., Bleicken, S., García-Sáez, A. J., Jeschke, G. & Meiler, J. (2016). *J. Struct. Biol.* **195**, 62–71. 10.1016/j.jsb.2016.04.014 PMC4912045 27129417

[bb28] Gatti, L. (2015). *Curr. Biotechnol.* **4**, 16–25.

[bb29] Göbl, C., Madl, T., Simon, B. & Sattler, M. (2014). *Prog. Nucl. Magn. Reson. Spectrosc.* **80**, 26–63. 10.1016/j.pnmrs.2014.05.003 24924266

[bb30] González, A. J., Liao, L. & Wu, C. H. (2013). *Bioinformatics*, **29**, 1018–1025. 10.1093/bioinformatics/btt076 PMC3624801 23418186

[bb31] Grigolon, S., Franz, S. & Marsili, M. (2016). *Mol. Biosyst.* **12**, 2147–2158. 10.1039/c6mb00047a 26974515

[bb32] Gueudré, T., Baldassi, C., Zamparo, M., Weigt, M. & Pagnani, A. (2016). *Proc. Natl Acad. Sci. USA*, **113**, 12186–12191. 10.1073/pnas.1607570113 PMC5087065 27729520

[bb33] Heffernan, R., Dehzangi, A., Lyons, J., Paliwal, K., Sharma, A., Wang, J., Sattar, A., Zhou, Y. & Yang, Y. (2015). *Bioinformatics*, **32**, 843–849. 10.1093/bioinformatics/btv665 26568622

[bb34] Hendrickson, W. A. (2014). *Q. Rev. Biophys.* **47**, 49–93. 10.1017/S0033583514000018 PMC4128195 24726017

[bb35] Hönigschmid, P. & Frishman, D. (2016). *J. Struct. Biol.* **194**, 112–123. 10.1016/j.jsb.2016.02.005 26851352

[bb36] Hopf, T. A., Colwell, L. J., Sheridan, R., Rost, B., Sander, C. & Marks, D. S. (2012). *Cell*, **149**, 1607–1621. 10.1016/j.cell.2012.04.012 PMC3641781 22579045

[bb37] Hopf, T. A., Ingraham, J. I., Poelwijk, F. J., Scharfe, C. P. I., Springer, M., Sander, C. & Marks, D. S. (2016). *Nature Biotechnol.* **35**, 128–135. 10.1038/nbt.3769 PMC5383098 28092658

[bb38] Hopf, T. A., Schärfe, C. P. I., Rodrigues, J. P. G. L. M., Green, A. G., Kohlbacher, O., Sander, C., Bonvin, A. M. J. J. & Marks, D. S. (2014). *Elife*, **3**, 10. 10.7554/eLife.03430 PMC4360534 25255213

[bb39] Iserte, J., Simonetti, F. L., Zea, D. J., Teppa, E. & Marino-Buslje, C. (2015). *Nucleic Acids Res.* **43**, W320–W325. 10.1093/nar/gkv572 PMC4489276 26032772

[bb40] Jana, B., Morcos, F. & Onuchic, J. N. (2014). *Phys. Chem. Chem. Phys.* **16**, 6496–6507. 10.1039/c3cp55275f 24603809

[bb41] Jeong, C. & Kim, D. (2016). *BMC Bioinformatics*, **17**, 99. 10.1186/s12859-016-0948-2 PMC4765150 26911566

[bb42] Jiménez-García, B., Pons, C., Svergun, D. I., Bernadó, P. & Fernández-Recio, J. (2015). *Nucleic Acids Res.* **43**, W356–W361. 10.1093/nar/gkv368 PMC4489248 25897115

[bb43] Johnson, L. S., Eddy, S. R. & Portugaly, E. (2010). *BMC Bioinformatics*, **11**, 431. 10.1186/1471-2105-11-431 PMC2931519 20718988

[bb44] Jones, D. T., Buchan, D. W. A., Cozzetto, D. & Pontil, M. (2012). *Bioinformatics*, **28**, 184–190. 10.1093/bioinformatics/btr638 22101153

[bb45] Jones, D. T., Singh, T., Kosciolek, T. & Tetchner, S. (2015). *Bioinformatics*, **31**, 999–1006. 10.1093/bioinformatics/btu791 PMC4382908 25431331

[bb46] Jones, S. & Thornton, J. M. (1996). *Proc. Natl Acad. Sci. USA*, **93**, 13–20.

[bb47] Joseph, A. P., Swapna, L. S., Rakesh, R. & Srinivasan, N. (2016). *J. Struct. Biol.* **195**, 294–305. 10.1016/j.jsb.2016.07.012 27444391

[bb48] Juan, D. de, Pazos, F. & Valencia, A. (2013). *Nat. Rev. Genet.* **14**, 249–261. 10.1038/nrg3414 23458856

[bb49] Kamisetty, H., Ovchinnikov, S. & Baker, D. (2013). *Proc. Natl Acad. Sci. USA*, **110**, 15674–15679. 10.1073/pnas.1314045110 PMC3785744 24009338

[bb50] Keegan, R., Waterman, D. G., Hopper, D. J., Coates, L., Taylor, G., Guo, J., Coker, A. R., Erskine, P. T., Wood, S. P. & Cooper, J. B. (2016). *Acta Cryst.* D**72**, 933–943. 10.1107/S2059798316010433 27487824

[bb51] Kelley, L. A., Mezulis, S., Yates, C. M., Wass, M. N. & Sternberg, M. J. (2015). *Nat. Protoc.* **10**, 845–858. 10.1038/nprot.2015.053 PMC5298202 25950237

[bb52] Koehler Leman, J., Ulmschneider, M. B. & Gray, J. J. (2015). *Proteins*, **83**, 1–24. 10.1002/prot.24703 PMC4270820 25355688

[bb53] Kosciolek, T. & Jones, D. T. (2015). *Proteins*, **84**, Suppl. 1, 145–151. 10.1002/prot.24863 PMC5042084 26205532

[bb54] Krissinel, E. (2015). *Nucleic Acids Res.* **43**, W314–W319. 10.1093/nar/gkv314 PMC4489313 25908787

[bb55] Lange, O. F., Rossi, P., Sgourakis, N. G., Song, Y., Lee, H. W., Aramini, J. M., Ertekin, A., Xiao, R., Acton, T. B., Montelione, G. T. & Baker, D. (2012). *Proc. Natl Acad. Sci. USA*, **109**, 10873–10878. 10.1073/pnas.1203013109 PMC3390869 22733734

[bb56] Langer, G., Cohen, S. X., Lamzin, V. S. & Perrakis, A. (2008). *Nat. Protoc.* **3**, 1171–1179. 10.1038/nprot.2008.91 PMC2582149 18600222

[bb57] Lapedes, A. S., Giraud, B. G., Liu, L. & Stormo, G. D. (1999). *Statistics in Molecular Biology and Genetics*, pp. 236–256. Hayward: Institute of Mathematical Statistics. https://doi.org/10.1214/lnms/1215455556.

[bb58] Ma, J., Wang, S., Wang, Z. & Xu, J. (2015). *Bioinformatics*, **31**, 3506–3513. 10.1093/bioinformatics/btv472 PMC4838177 26275894

[bb59] Marks, D. S., Colwell, L. J., Sheridan, R., Hopf, T. A., Pagnani, A., Zecchina, R. & Sander, C. (2011). *PLoS One*, **6**, e28766. 10.1371/journal.pone.0028766 PMC3233603 22163331

[bb60] Marks, D. S., Hopf, T. A. & Sander, C. (2012). *Nat. Biotechnol.* **30**, 1072–1080. 10.1038/nbt.2419 PMC4319528 23138306

[bb61] Morcos, F., Pagnani, A., Lunt, B., Bertolino, A., Marks, D. S., Sander, C., Zecchina, R., Onuchic, J. N., Hwa, T. & Weigt, M. (2011). *Proc. Natl Acad. Sci. USA*, **108**, E1293–E1301. 10.1073/pnas.1111471108 PMC3241805 22106262

[bb62] Nicoludis, J. M., Lau, S.-Y., Schärfe, C. P. I., Marks, D. S., Weihofen, W. A. & Gaudet, R. (2015). *Structure*, **23**, 2087–2098. 10.1016/j.str.2015.09.005 PMC4635037 26481813

[bb63] Ochoa, D. & Pazos, F. (2010). *Bioinformatics*, **26**, 1370–1371. 10.1093/bioinformatics/btq137 20363731

[bb64] Oliveira, S. H. de, Shi, J. & Deane, C. M. (2016). *Bioinformatics*, **33**, 373–381.

[bb65] Ovchinnikov, S., Kamisetty, H. & Baker, D. (2014). *Elife*, **3**, e02030. 10.7554/eLife.02030 PMC4034769 24842992

[bb66] Ovchinnikov, S., Kim, D. E., Wang, R. Y., Liu, Y., DiMaio, F. & Baker, D. (2015). *Proteins*, **84**, Suppl. 1, 67–75. 10.1002/prot.24974 PMC5490371 26677056

[bb67] Ovchinnikov, S., Kinch, L., Park, H., Liao, Y., Pei, J., Kim, D. E., Kamisetty, H., Grishin, N. V. & Baker, D. (2015). *eLife*, **4**, e09248. 10.7554/eLife.09248 PMC4602095 26335199

[bb68] Ovchinnikov, S., Park, H., Varghese, N., Huang, P.-S., Pavlopoulos, G. A., Kim, D. E., Kamisetty, H., Kyrpides, N. C. & Baker, D. (2017). *Science*, **355**, 294–298. 10.1126/science.aah4043 PMC5493203 28104891

[bb69] Pandurangan, A. P., Vasishtan, D., Alber, F. & Topf, M. (2015). *Structure*, **23**, 2365–2376. 10.1016/j.str.2015.10.013 PMC4671957 26655474

[bb70] Parente, D. J., Ray, J. C. & Swint-Kruse, L. (2015). *Proteins*, **83**, 2293–2306. 10.1002/prot.24948 PMC4715571 26503808

[bb71] Raman, S., Lange, O. F., Rossi, P., Tyka, M., Wang, X., Aramini, J., Liu, G., Ramelot, T. A., Eletsky, A., Szyperski, T., Kennedy, M. A., Prestegard, J., Montelione, G. T. & Baker, D. (2010). *Science*, **327**, 1014–1018. 10.1126/science.1183649 PMC2909653 20133520

[bb72] Ramírez-Aportela, E., López-Blanco, J. R. & Chacón, P. (2016). *Bioinformatics*, **32**, 2386–2388. 10.1093/bioinformatics/btw141 27153583

[bb73] Remmert, M., Biegert, A., Hauser, A. & Söding, J. (2011). *Nat. Methods*, **9**, 173–175. 10.1038/nmeth.1818 22198341

[bb74] Rigden, D. J. (2002). *Protein Eng.* **15**, 65–77. 10.1093/protein/15.2.65 11917143

[bb75] Rigden, D. J. (2017). Editor. *From Protein Structure to Function with Bioinformatics*, 2nd ed. Heidelberg: Springer Nature.

[bb76] Rupp, B. (2009). *Biomolecular Crystallography: Principles, Practice, and Application to Structural Biology*, p. 627. New York: Garland Science.

[bb77] Sadowski, M. I. (2013). *Proteins*, **81**, 253–260. 10.1002/prot.24181 PMC3563215 22987736

[bb78] Safarian, S., Rajendran, C., Müller, H., Preu, J., Langer, J. D., Ovchinnikov, S., Hirose, T., Kusumoto, T., Sakamoto, J. & Michel, H. (2016). *Science*, **352**, 583–586. 10.1126/science.aaf2477 PMC5515584 27126043

[bb79] Sammito, M., Millán, C., Rodríguez, D. D., de Ilarduya, I. M., Meindl, K., De Marino, I., Petrillo, G., Buey, R. M., de Pereda, J. M., Zeth, K., Sheldrick, G. M. & Usón, I. (2013). *Nat. Methods*, **10**, 1099–1101. 10.1038/nmeth.2644 24037245

[bb80] Schep, D. G., Zhao, J. & Rubinstein, J. L. (2016). *Proc. Natl Acad. Sci. USA*, **113**, 3245–3250. 10.1073/pnas.1521990113 PMC4812769 26951669

[bb81] Schindler, C. E., de Vries, S. J., Sasse, A. & Zacharias, M. (2016). *Structure*, **24**, 1387–1397. 10.1016/j.str.2016.06.007 27427479

[bb82] Schot, G. van der & Bonvin, A. M. J. J. (2015). *J. Biomol. NMR*, **62**, 497–502. 10.1007/s10858-015-9942-7 PMC4569659 25982706

[bb83] Seemayer, S., Gruber, M. & Söding, J. (2014). *Bioinformatics*, **30**, 3128–3130. 10.1093/bioinformatics/btu500 PMC4201158 25064567

[bb84] Segura, J., Sanchez-Garcia, R., Tabas-Madrid, D., Cuenca-Alba, J., Sorzano, C. O. & Carazo, J. M. (2016). *Biophys. J.* **110**, 766–775. 10.1016/j.bpj.2015.11.3519 PMC4775853 26772592

[bb85] Sfriso, P., Duran-Frigola, M., Mosca, R., Emperador, A., Aloy, P. & Orozco, M. (2016). *Structure*, **24**, 116–126. 10.1016/j.str.2015.10.025 26688214

[bb86] Shackelford, G. & Karplus, K. (2007). *Proteins*, **69**, Suppl. 8, 159–164. 10.1002/prot.21791 17932918

[bb87] Simkovic, F., Thomas, J. M. H., Keegan, R. M., Winn, M. D., Mayans, O. & Rigden, D. J. (2016). *IUCrJ*, **3**, 259–270. 10.1107/S2052252516008113 PMC4937781 27437113

[bb111] Simkovic, F., Thomas, J. M. & Rigden, D. J. (2017). *Bioinformatics*, https://doi.org/10.1093/bioinformatics/btx148.

[bb88] Skerker, J. M., Perchuk, B. S., Siryaporn, A., Lubin, E. A., Ashenberg, O., Goulian, M. & Laub, M. T. (2008). *Cell*, **133**, 1043–1054. 10.1016/j.cell.2008.04.040 PMC2453690 18555780

[bb89] Skwark, M. J., Raimondi, D., Michel, M. & Elofsson, A. (2014). *PLoS Comput. Biol.* **10**, e1003889. 10.1371/journal.pcbi.1003889 PMC4222596 25375897

[bb90] Slabinski, L., Jaroszewski, L., Rychlewski, L., Wilson, I. A., Lesley, S. A. & Godzik, A. (2007). *Bioinformatics*, **23**, 3403–3405. 10.1093/bioinformatics/btm477 17921170

[bb91] Söding, J., Biegert, A. & Lupas, A. N. (2005). *Nucleic Acids Res.* **33**, W244–W248. 10.1093/nar/gki408 PMC1160169 15980461

[bb92] Stokes-Rees, I. & Sliz, P. (2010). *Proc. Natl Acad. Sci. USA*, **107**, 21476–21481. 10.1073/pnas.1012095107 PMC3003117 21098306

[bb93] Svergun, D. I., Koch, M. H. J., Timmins, P. A. & May, R. P. (2013). *Small Angle X-ray and Neutron Scattering from Solutions of Biological Macromolecules.* Oxford University Press.

[bb94] Tang, Y., Huang, Y. J., Hopf, T. A., Sander, C., Marks, D. S. & Montelione, G. T. (2015). *Nat. Methods*, **12**, 751–754. 10.1038/nmeth.3455 PMC4521990 26121406

[bb95] Taylor, W. R. (2016). *Algorithms Mol. Biol.* **11**, 17. 10.1186/s13015-016-0080-x PMC4912788 27330543

[bb96] Torchala, M., Moal, I. H., Chaleil, R. A., Fernandez-Recio, J. & Bates, P. A. (2013). *Bioinformatics*, **29**, 807–809. 10.1093/bioinformatics/btt038 23343604

[bb97] Toth-Petroczy, A., Palmedo, P., Ingraham, J., Hopf, T. A., Berger, B., Sander, C. & Marks, D. S. (2016). *Cell*, **167**, 158–170.e12. 10.1016/j.cell.2016.09.010 PMC5451116 27662088

[bb98] UniProt Consortium (2015). *Nucleic Acids Res.* **43**, D204–D212. 10.1093/nar/gku989 PMC4384041 25348405

[bb99] Wang, Y. & Barth, P. (2015). *Nat. Commun.* **6**, 7196. 10.1038/ncomms8196 PMC4833009 25995083

[bb100] Wang, R. Y., Kudryashev, M., Li, X., Egelman, E. H., Basler, M., Cheng, Y., Baker, D. & DiMaio, F. (2015). *Nat. Methods*, **12**, 335–338. 10.1038/nmeth.3287 PMC4435692 25707029

[bb101] Wang, S., Sun, S., Li, Z., Zhang, R. & Xu, J. (2017). *PLoS Comput. Biol.* **13**, e1005324. 10.1371/journal.pcbi.1005324 PMC5249242 28056090

[bb102] Wang, Z. & Xu, J. (2013). *Bioinformatics*, **29**, 266–273.

[bb103] Webb, B., Lasker, K., Schneidman-Duhovny, D., Tjioe, E., Phillips, J., Kim, S. J., Velázquez-Muriel, J., Russel, D. & Sali, A. (2011). *Methods Mol. Biol.* **781**, 377–397. 10.1007/978-1-61779-276-2_19 21877292

[bb104] Wuyun, Q., Zheng, W., Peng, Z. & Yang, J. (2016). *Brief Bioinform.*, https://doi.org/10.1093/bib/bbw106.

[bb105] Xu, D., Jaroszewski, L., Li, Z. & Godzik, A. (2014). *Bioinformatics*, **30**, 660–667. 10.1093/bioinformatics/btt578 PMC3933871 24130308

[bb106] Yang, J., Jin, Q.-Y., Zhang, B. & Shen, H.-B. (2016). *Bioinformatics*, **32**, 2435–2443. 10.1093/bioinformatics/btw181 27153618

[bb107] Yu, J., Vavrusa, M., Andreani, J., Rey, J., Tufféry, P. & Guerois, R. (2016). *Nucleic Acids Res.* **44**, W542–W549. 10.1093/nar/gkw340 PMC4987904 27131368

[bb108] Zacharchenko, T., von Castelmur, E., Rigden, D. J. & Mayans, O. (2015). *Biochem. Soc. Trans.* **43**, 850–855. 10.1042/BST20150084 26517893

[bb109] Zhang, H., Huang, Q., Bei, Z., Wei, Y. & Floudas, C. A. (2016). *Proteins*, **84**, 332–348. 10.1002/prot.24979 26756402

[bb110] Zimmerman, B., Kelly, B., McMillan, B. J., Seegar, T. C., Dror, R. O., Kruse, A. C. & Blacklow, S. C. (2016). *Cell*, **167**, 1041–1051. 10.1016/j.cell.2016.09.056 PMC5127602 27881302

